# Peptic Ulcer Disease among Patients Undergoing Upper Gastrointestinal Endoscopy in a Tertiary Care Centre: A Descriptive Cross-sectional Study

**DOI:** 10.31729/jnma.8527

**Published:** 2024-04-30

**Authors:** Shova Sapkota, Saurav Sen Oli, Mamata Karki, Prerana Singh Rokaha, Anant Aryal

**Affiliations:** 1 Kathmandu Medical College and Teaching Hospital, Sinamangal, Kathmandu, Nepal; 2 Lumbini Medical College and Teaching Hospital, Tansen, Palpa, Nepal; 3 Department of Internal Medicine, Kathmandu Medical College and Teaching Hospital, Sinamangal, Kathmandu, Nepal

**Keywords:** *digestive system endoscopy*, *duodenal ulcer*, *stomach ulcer*

## Abstract

**Introduction:**

Peptic ulcer is a common disease of gastrointestinal tract usually present with epigastric pain and discomfort. Upper gastrointestinal endoscopy is its gold standard investigation. There has been limited study on the prevalence of peptic ulcer disease among patients undergoing upper GI endoscopy especially in Nepal. Our study aimed to find the prevalence of peptic ulcer disease among patients undergoing upper GI endoscopy at our center.

**Methods:**

A descriptive cross-sectional study was conducted among patients undergoing upper gastrointestinal endoscopy at a tertiary care center from October 1, 2022 to March 31, 2023. Data was retrieved from hospital records using a preformed proforma and sample size of 219 was calculated and data of 273 cases was collected using the convenience method of sampling.

**Results:**

Among 273 patients, peptic ulcer disease was found in 29 (10.62%) of patients among which 28 (10.25%) had antral ulcer and only 1 (0.36%) had duodenal ulcer.

**Conclusions:**

The prevalence of peptic ulcer disease is lower in our study center compared to other studies and further studies can be conducted on the associated risk factors and socio-demographic distribution of peptic ulcer disease.

## INTRODUCTION

Peptic ulcer disease (PUD) is defined as disruption in gastrointestinal (GI) tract epithelium extending upto muscularis propria and usually involves stomach and proximal duodenum but may extend upto the lower esophagus, distal duodenum, or jejunum.^1^ PUD is a common disease with prevalence of 5-15% among adult population of world.^2,3^

Patient usually present with epigastric pain, discomfort, anorexia and weight loss and has complications like upper GI bleeding, perforation or gastric outlet obstruction.^4^ Helicobacter pylori infection and Nonsteroidal anti-inflammatory drugs (NSAID) use are the major cause of PUD, which is still high in developing country whereas emerging antimicrobial resistance pose a therapeutic challenge.^5,6^ Though PUD is a common disease presentation in gastro medicine OPD, there has been limited study on its prevalence among patients undergoing upper GI endoscopy.

In Nepal our study aimed to find the prevalence of peptic ulcer disease among patients undergoing upper GI endoscopy at center.

## METHODS

This study was a descriptive cross-sectional study conducted among adults undergoing upper gastrointestinal endoscopy at a tertiary care center, Kathmandu Medical College Teaching Hospital, Nepal. We retrieved the medical records of all the patients who presented with gastrointestinal symptoms and underwent upper GI endoscopy from October 1 2022 to March 31 2023. Ethical clearance was obtained from the Institutional Review Board (Reference number: 21042023/06). No identifiable individual information was used and patient records were removed before being included in the analysis. Adult patients presenting with gastrointestinal symptoms undergoing upper GI endoscopy during the study period were included in the study. Convenience sampling was done. The sample size was calculated using the formula:


$$\begin{array}{ll} \text{n} & ={\text{Z}}^{2}\times \frac{\text{p}\times \text{q}}{{\text{e}}^{\text{2}}}\\ & =1.96^{2}\times \frac{0.172\times 0.828}{0.05^{2}}\\ & =218.842 \end{array}$$

Where,

n = required sample size
Z = 1.96 at 95% Confidence Interval (CI)
p = prevalence, 17.2% - prevalence of peptic ulcer disease among patients undergoing upper GI endoscopy.^7^
e = margin of error, 5%

So, records of the upper GI endoscopy from Oct 1, 2022 to 31st March 2023 which was 273 cases were taken, 54 cases more than the sample size calculated. Documents and medical records of the patient undergoing upper GI endoscopy were assessed for personal data, medical history and endoscopy findings. We collected data according to the performed proforma.

Data were entered using the Microsoft Excel and Statistical Package for the Social Sciences (SPSS) software was used for analysis. Point estimate at 95% Confidence Interval was calculated.

## RESULTS

Among 273 patients, where 128 (46.90%) of them were male and 145 (53.10%) of them were female, peptic ulcer disease was detected in 29 (10.62%) of patients undergoing upper GI endoscopy. Out of the 29 patients diagnosed with peptic ulcers, 15 (51.72%) were female, and 14 (48.27%) were male, with a confidence interval of 95%.

**Table 1:** Endoscopy finding in patients undergoing upper gastrointestinal endoscopy (n= 29).

Endoscopy findings	n (%)
Antral Ulcer	28 (10.26)
Forest I	5 (1.83)
Forest II	8 (2.93)
Forest III	15 (5.49)
Duodenal Ulcer	1 (0.37)

**Table 2:** Age wise distribution of patients diagnosed with peptic ulcer in upper gastrointestinal endoscopy (n= 29).

Age group (years)	n (%)
20-29	3 (10.34)
30-39	15 (51.72)
40-49	8 (27.59)
50-59	2 (6.90)
60-69	1 (3.45)

## DISCUSSION

Among 273 individuals who had upper GI endoscopy in our study, 29 (10.62%) had peptic ulcer disease (Table 1). Similar research on gastrointestinal endoscopies on 2735 symptomatic patients in the Van region revealed a somewhat greater prevalence of peptic ulcer disease 298 (10.9%) in those patients than in our study.^9^ Similarly there are other studies with higher prevalence of peptic ulcer disease than of ours.^7,8,10^ In another similar study, peptic ulcer disease was found to be significantly lower where it was 27 (8.47%) of symptomatic patients undergoing upper GI endoscopy.^6^ Also in our study, females had a slightly greater proportion of peptic ulcer disease than males: 15 (51.72%) vs 14 (48.27%). Unlike our study proportion of male is higher than female in other studies.^6-13^

The age range of 30-39 years, which accounted for 15 (51.72%) of all cases, showed a high frequency of PUD (Table 2). In some other similar studies peptic ulcer disease is most commonly seen in the age group of 40-50 years.^9,11^ Work, busy schedule, as well as inappropriate and irregular eating habits may be the reason for high prevalence of peptic ulcer in middle aged people.

Out of 29 peptic ulcers, 28 (96.55%) had antral ulcer and only 1 (3.45%) had duodenal ulcer. Similar to our study, gastric ulcer occurred higher than duodenal ulcer in a study among asymptomatic patient under upper GI endoscopy.^12^ In contrast to our study, many of the studies showed higher prevalence of duodenal ulcers than gastric ulcer.^9,11^

A number of studies carried out over the previous 2030 years have shown a sharp decline in the prevalence of PUD, PUD-related hospital admissions, and PUD-associated mortality as a result of the use of new antiPUD therapies, such as the removal of *Helicobacter pylori(H. pylori)* and the use of proton-pump inhibitors (PPIs).^14^ The picture of PUD has evolved recently, though, as increased physiological stress, histamine 2 receptor antagonists, and selective serotonin reuptake medications, along with their widespread use, have been identified as risk factors.^14^

Since the study is done only in a single medical institution and in a single nation, this research cannot be generalized to all the patients of other places. Also, as convenience sampling was used, selection bias and sampling bias was unavoidable.

## CONCLUSIONS

Our study demonstrated a relatively lower proportion of peptic ulcer among patients undergoing upper GI endoscopy. In our study, duodenal ulcer is lower than gastric ulcer and proportion of ulcer is slightly more in females than in males with 30-39 age group accounting for high frequency of peptic ulcer. Further studies can be conducted on the associated risk factors and sociodemographic distribution of PUD.
